# Comparison of the Minimental State Examination Scale and the International HIV Dementia Scale in Assessing Cognitive Function in Nigerian HIV Patients on Antiretroviral Therapy

**DOI:** 10.1155/2012/581531

**Published:** 2012-09-25

**Authors:** O. Olajumoke Oshinaike, A. Akinsegun Akinbami, O. Oluwadamilola Ojo, I. Frank Ojini, U. Njideka Okubadejo, A. Mustapha Danesi

**Affiliations:** ^1^Department of Medicine, College of Medicine, Lagos State University, Nigeria; ^2^Department of Hematology and Blood Transfusion, College of Medicine, Lagos State University, Nigeria; ^3^College of Medicine, University of Lagos, Lagos State, Idi-Araba, Nigeria

## Abstract

*Introduction*. HIV-associated neurocognitive disorder (HAND) remains common despite the availability of antiretroviral therapy. Routine screening will improve early detections. *Objective*. To compare the performance of the minimental state examination (MMSE) and international HIV dementia scale (IHDS) in assessing neurocognitive function in HIV/AIDS patients on antiretroviral therapy. *Methods*. A case-control study of 208 HIV-positive and 121 HIV-negative individuals. Baseline demographic data were documented and cognitive function assessed using the two instruments. CD4 cell counts were recorded. *Results*. Cases comprised 137 females and 71 males. Controls were 86 females and 35 males. Mean MMSE score of cases was 27.7 ± 1.8 compared to 27.8 ± 1.3 in controls (*P* = 0.54). Mean IHDS score in cases was 8.36 ± 3.1 compared to 10.7 ± 0.9 in controls (*P* < 0.001). Using the MMSE scale, 6 cases but no controls had HAND (*P* = 0.09). Using the IHDS, 113 (54.3%) had HAND compared with 10 (8.3%) controls (*P* < 0.0001). Using IHDS, 56.5% cases with CD4 count > 200 had HAND compared with 92.5% with CD4 count < 200 (*P* < 0.001). *Conclusion*. These findings indicate that the IHDS detects higher rates of HAND and may identify HIV/AIDS patients who require further cognitive assessment using more robust assessment batteries.

## 1. Introduction

HIV-associated neurocognitive disorder is often encountered in HIV infection despite the use of potent antiretroviral therapy. The spectrum ranges from mild and asymptomatic neurocognitive impairment (ANI), minor neurocognitive disorder (MND), to the more severe HIV-associated dementia (HAD) [1]. ANI is characterized by asymptomatic or unrecognized neurocognitive impairment that may go unnoticed except specifically screened for, and individuals with ANI are more likely to progress to more severe forms of cognitive dysfunction. The essential features of MND are impaired cognitive or behavioral function in at least 2 domains (e.g., impaired attention-concentration, mental slowing, abnormal memory or other cognitive functions, slowed movements, incoordination, personality change, irritability, and emotional lability). In contrast to ANI, these abnormalities typically impair work-related function or activities of daily living, albeit mildly. MND is associated with shortened survival, reduced adherence with antiretroviral therapy, and problems with employability, and its presence is predictive of HAD. HAD represents the most severe form of cognitive dysfunction, with significant functional impairments, and is synonymous with HIV encephalopathy and AIDS dementia complex (ADC). ADC is one of the most common central nervous system complications of late HIV infection occurring in 15–20% of patients before the introduction of HAART [2, 3]. 

The widespread use of HAART has resulted in a sharp decline in its incidence but the prevalence has actually increased because of prolonged survival [4–6]. Similarly, the prevalence of minor cognitive deficits appears to have increased, with reported prevalence between 20 and 50% of HIV-positive patients [4, 5, 7–9]. The prevalence of cognitive impairment in the aviremic HIV-positive population was 69% in one study [10]. Risk factors for HAND in HIV include a high HIV viral setpoint, lower CD4 cell counts, anemia, low body mass index, increasing age, systemic symptoms, injection drug use, and female gender [11–14].

Screening for early deficits and careful evaluation of psychomotor function would permit the use of additional treatments [15–17] to improve cognitive functioning. The diagnosis of HAND in HIV is dependent upon a clinical history and neurological examination consistent with the criteria developed by the American Academy of Neurology [18]. Neuropsychological testing is a critical component of the diagnosis showing abnormalities in psychomotor speed, attention, frontal lobe function, and verbal and nonverbal memory. However, administration of the entire neuropsychological test battery is cumbersome in a real-world clinical scenario because it is time-consuming, language and education dependent, and manpower intensive. In most countries of sub-Saharan Africa where the vast majority of HIV cases reside, simpler but effective screening tools are required to enhance early recognition of persons with cognitive dysfunction. The ideal screening tool should emphasize motor skills and timed tasks, must be inexpensive, universally available, brief, sensitive, and reliable. The Minimental state examination scale (MMSE) is a generic instrument that was originally developed to screen for dementia and delirium and is the most widely used cognitive impairment screening instrument. Despite its ease of administration and wide recognition, the validity of the MMSE in subcortical disorders such as HIV-associated cognitive impairment has been criticized. In the pre-HAART era, the HIV dementia scale (HDS) was developed [19] and subsequently modified for use in international settings as the International HIV Dementia Scale (IHDS) [20]. The study hypothesis was that there is a significant difference in the detection of HAND when MMSE is used compared to the IHDS scale. Specifically, we predicted that the IHDS would identify more cases of HIV with HAND compared with MMSE. Our purpose was to either buttress or refute the current practice of using the MMSE instead of the IHDS scale in the setting of HIV/AIDS.

## 2. Methodology

### 2.1. Study Setting and Design

The HIV clinic at the Lagos University Teaching Hospital (LUTH) is one of several HIV follow-up and treatment centers in the country funded by the Presidents Emergency Programme for AIDS Relief in Africa (PEPFAR) and is a referral center attending to about 9,000 patients annually. We used a case-control study design involving HIV-positive adults (aged >18 years) as cases and HIV-negative age-matched adults as controls. Approval of the study protocol was obtained from the Health Research and Ethics Committee of the LUTH. Informed consent was obtained from all participants.

### 2.2. Participant Recruitment and Data Collection

The HIV-positive cases were recruited consecutively over a 12-month period between June 2007 and May 2008. HIV-negative controls subjects were recruited from the HIV voluntary counseling and testing section of the same hospital. Cases were matched for age and sex with the controls. All cases included had low CD4 cell counts and had been on antiretroviral therapy for at least 6 months. Exclusion criteria were major opportunistic infections of the brain in the past 3 years, any other opportunistic infection not affecting the brain in the past 12 months (very ill patients), major depression according to Diagnostic and Statistical Manual of Mental Disorders, 4th edition criteria, active injection/inhalational recreational drug use (e.g., IV heroin, marijuana), and pregnancy. 

Baseline demographic parameters, medical history, physical and neurological examination were documented using a standardized proforma. For all HIV-positive cases, latest (within preceding 3 months) CD4 count result was extracted from the clinic electronic database or case records. Face-to-face neuropsychological testing was conducted using both the MMSE [21] and the IHDS in each participant. The interviews were done by a trained neurologist conversant with the application of both instruments (O. O. Oshinaike).

### 2.3. Description of Study Instruments

The IHDS is a modification of the HIV dementia scale first proposed by Power et al. (1995) [19] and recently adapted by Sacktor et al. (2005) [20]. It consists of 3 subsets: timed finger tapping which measures motor speed; timed alternating hand sequence which assesses the psychomotor speed; recall of 4 items in 2 minutes which assesses memory registration and recall. Each of these subtests is rated on a scale of 0–4. The tests were administered as follows: for assessment of the verbal recall subtest, registration (new learning) was measured by reciting 4 words to the subject (blue, dog, hat, and apple) taking 1 second to say each of the words. The subject was asked to repeat the words and recall the 4 words after the timed finger tapping, and alternating hand sequence tests were performed. The MMSE is an interviewer-administered questionnaire testing 5 domains (orientation, memory registration, attention and calculation, memory recall, and language) with a maximum score of 30 points. The cut-off values for defining cognitive impairment using the MMSE and IHDS, respectively, were 26 and 10 (based on the mean score for controls minus 1 standard deviation).

### 2.4. Data Analysis

Data entry and analysis were achieved using Epi Info (Epi 3.5.1 version) statistical software. Group differences in mean values of numerical data (including age, CD4 cell count, and test scores) were compared using Student's *t*-test, while Pearson Chi-square was used to determine statistical significance of group differences for categorical variables. Level of significance was set at *P* value < 0.05. 

## 3. Results

### 3.1. Demographic Characteristics

The 208 cases comprised 137 (65.9%) females and 71 (34.1%) males with a mean age ± SD of 36.8 ± 8.3 years (range 19–63 years). The control group (total number = 121) was made up of 86 (71.1%) females and 35 (28.9%) males, with a mean age of 38.0 ± 8.4 years (range 22–66 years). The age and gender differences were not statistically significant (*P* = 0.18 and 0.33 resp.). The mean CD4 count of cases was 251.4 ± 171.4 cells/mm^3^ (range 4–939 cells/mm^3^). Data shown in Table 1. 

### 3.2. Comparison of Cognitive Performance Using the IHDS and the MMSE Scores

**Figure 1:**
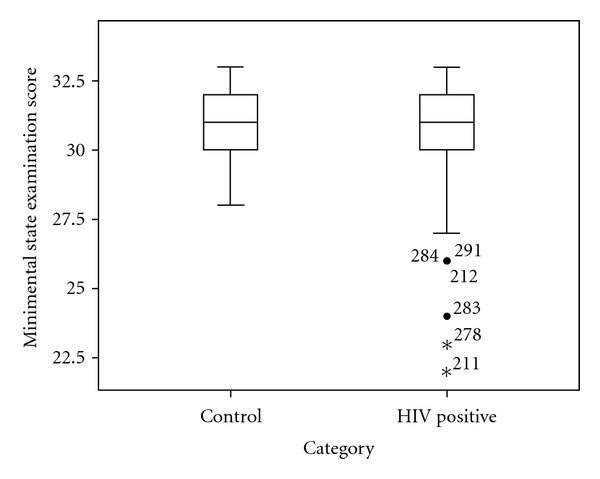
Comparison of MMSE scores in HIV-positive cases and HIV-negative controls. Box plot illustrating the distribution of MMSE scores in cases and controls. The mean ± SD) MMSE score of the controls (27.8 ± 1.3) and HIV-positive cases (27.7 ± 1.8) did not differ significantly (ANOVA; *P* = 0.59). Asterisked cases represent outliers within the HIV-positive group with MMSE scores below the group minimum.

**Figure 2:**
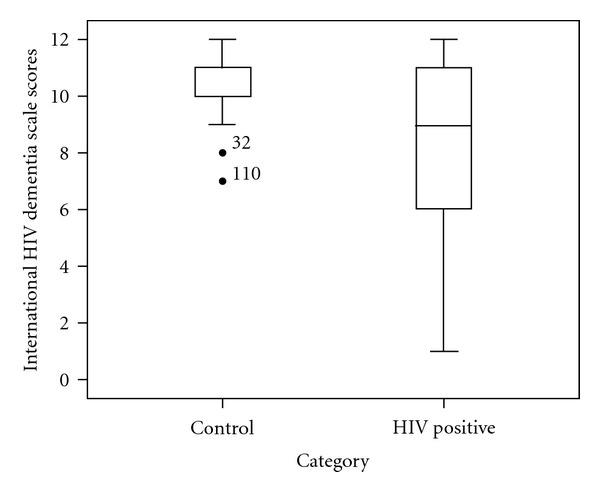
Comparison of IHDS scores in HIV-positive cases and HIV-negative controls. Box plot illustrating the distribution of IHDS scores in cases and controls. The mean (SD) score of the controls (10.7 ± 0.9) and HIV-positive cases (8.36 ± 3.1) differed significantly (ANOVA; *P* < 0.0001). Asterisked cases represent outliers within the control group with scores below the group minimum.

**Figure 3:**
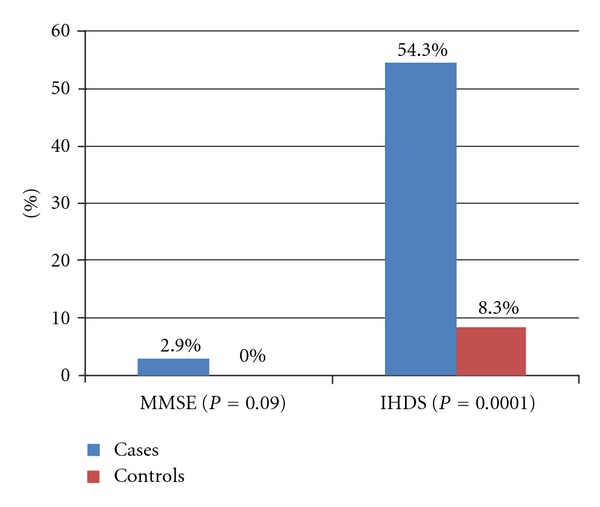
Frequency of cognitive impairment based on the MMSE and IHDS scores of cases (HIV-positive) and controls (HIV-negative).

The mean MMSE scores of HIV-positive cases was 27.7 ± 1.8 compared with 27.8 ± 1.3 for controls (*P* = 0.54) whilst the mean score of cases using the IHDS scale was 8.36 ± 3.1 compared with 10.7 ± 0.9 in controls (*P* = 0.0001) (Figures 1 and 2). Based on the cut-off score of 26 to define HAND, Figure 3 shows that using the MMSE scale, 6 (2.9%) HIV cases were identified to have HAND compared to none of the controls (Fisher exact *P* = 0.09). Using the IHDS scale, based on a cut-off score of 10 (<1 SD below mean score of controls of 10.66) to define HAND, 113 HIV-positive cases (54.3%) were found to have HAND compared with 10 (8.3%) among the controls (*X* ^2^ = 69.3; *P* < 0.0001), whereas the MMSE did not detect any significant difference between HIV cases and controls, the IHDS showed a significantly higher frequency of HAND in cases. Furthermore, HAND detection rates within the HIV-positive group were significantly higher using the IHDS (113/208 i.e., 54.3%) compared to the MMSE (6/208 i.e., 2.9%) (odds ratio 0.02; 95% confidence interval 0.01–0.06; *P* < 0.0001).

### 3.3. Relationship of Disease Severity with Cognitive Scores

CD4 cell count (mm^3^) was categorized into two to reflect disease severity (defining severe disease as CD4 count ≤200). The study comprised 115 (55.3%) with CD4 count above 200 and 93 (44.7%) ≤ 200. Using the MMSE scale, 1 (0.9%) of cases with CD4 count >200 cells/mm^3^ had HAND compared with 5 (5.4%) of those with CD4 count ≤200 cells/mm^3^ (Fisher's exact *P* = 0.06). Conversely, with the IHDS scale, 44/115 (38.3%) of cases with CD4 count >200 cells/mm^3^ had HAND compared with 69/93 (74.2%) with CD4 count <200 cells/mm^3^ (*X* ^2^ = 26.8; *P* < 0.0001).

### 3.4. Classification of HAND according to Modified Updated AAN Criteria

There was insufficient data to credibly classify the subjects using these criteria (due to absence of data relating to impact on ADL/daily functioning). However with modification of the criteria, ANI and MND were grouped together (applying only the data regarding 1SD below control values and exclusion criteria) and HAD as any with values below 2SD. A total of 25 (12%) of cases had ANI and MND whilst 88 (42.3%) had HAD. The mean CD4 count of cases with ANI/MND was 208.4 99.7 cells/mm^3^ (median 188.0) whilst the mean CD4 count of cases with HAD was 173.4 1226 cells/mm^3^ (median 150.5) *P* = 0.0001. A total of 13/25 cases (52%) of ANI/MND had CD4 count <200 compared with 56/88 (63.6%) of cases with HAD *P* = 0.0001.

## 4. Discussion

There are few studies corroborating the superiority of the International HIV Dementia Scale over the MiniMental State Examination for assessment of HAND in persons with HIV via direct comparative studies and employing a control group. The main findings from our study are that the IHDS detects a higher proportion of persons with HAND in HIV, affording an advantage for more intensive evaluation and early interventions to improve quality of life. Although extensive neuropsychological testing using a combination of tests is regarded as the “gold standard” for cognitive assessment, the IHDS offers an advantage in the “real-world” clinical setting due to the ease of administration and can thus serve as an indicator of the need for further assessment and also serve as a monitoring tool in routine practice. The MMSE scale was only able to weakly distinguish HIV cases from controls with respect to occurrence of HAND. This reinforces previous observations alluding to its lack of sensitivity to sub-cortical cognitive dysfunction. Skinner et al. compared the performance of the original HIV dementia scale (HDS), IHDS, and MMSE scales against other neuro-cognitive batteries in assessing cognitive dysfunction in HIV patients and also demonstrated the inferiority of MMSE in contrast to the HDS and IHDS. This may be explained by the ability of the IHDS to screen for psychomotor speed (in addition to attention/working memory, executive functioning, memory, and verbal/language), an aspect that is not included in the MMSE scale. Also, literacy level and language comprehension impair the MMSE, thus further limiting its application. The low mean test scores in the control groups may be due to bias as MMSE scores have been shown to be affected by age, sex, lower education level and sociocultural background thereby leading to improper classification of individuals [22]. 

This study also demonstrated a statistically significantly higher frequency of HAND in relation to disease severity, with higher rates in HIV cases with more severe immune compromise (CD4 cell count below 200). The magnitude of the difference was also higher with the IHDS. This is explicable as CD4-related inflammatory changes in the brains of presymptomatic subjects are known to be mediated by the HIV-associated breakdown of the immune system and consequent lymphocyte dysfunction, allowing brain damage to occur. Also, the evidence for viral replication in the CNS, despite the lack of symptoms, suggests that neurocognitive functioning is likely to be more affected when more systemic immune suppression appears [23]. We noted a significantly lower mean CD4 cell count and disease severity in cases with HAD compared to ANI/MND using a modified classification of the levels of HAND according to the updated AAN criteria. 

Njamnshi et al. [24] reported a significant difference in mean HDS scores in HIV cases compared to HIV-negative controls. Lyon et al. [25] and Ganasen et al. [26] compared the HDS with the MMSE and found a higher sensitivity of 83% and 80%, respectively, using the HDS compared to 50% using the MMSE. 

## 5. Conclusion

Our study has added to the body of evidence encouraging the use of the IHDS as a screening instrument in the real-world clinical scenario of HIV/AIDS management, and we reemphasize that its intrinsic ability to reveal even mild cognitive impairment amenable to earlier intervention more than compensates for the possibility of a lower specificity. Incorporation of variables to determine affectation of activities of daily living into the original design of these instruments may assist in proper classification of patients especially in regions where more robust batteries are unavailable. With the accumulating evidence that standard HAART regimens are unable to fully reverse HAND and the unclear benefits of CNS-penetrant antiretroviral drugs even in the setting of long-term plasma viral suppression, there is a need for randomized prospective trials to explore the role of other adjuvant and neuroprotective therapies.

## Figures and Tables

**Table 1:** Baseline data and cognitive scores for participants in the study.

Variables	HIV-positive (cases) *N* = 208 (%)	HIV-negative (controls) *N* = 121 (%)	*P* value
Gender			
Male	71 (34.1%)	35 (28.9%)	0.39
Female	137 (65.9%)	86 (71.1%)	
Mean age (years)	36.8 ± 8.3	38.0 ± 8.4	0.21
Mean CD4 count ± SD (cells/mm^3^)	257.2	N/A	
Mean score MMSE ± SD	27.7 ± 1.8	27.8 ± 1.8	0.54
Mean score IHIDS ± SD	8.36 ± 3.1	10.7 ± 0.9	0.0001

N/A: not applicable.
